# DeepN4: Learning N4ITK Bias Field Correction for T1-weighted Images

**DOI:** 10.21203/rs.3.rs-3585882/v1

**Published:** 2023-11-13

**Authors:** Praitayini Kanakaraj, Tianyuan Yao, Leon Y. Cai, Ho Hin Lee, Nancy R. Newlin, Michael E. Kim, Chenyu Gao, Kimberly R. Pechman, Derek Archer, Timothy Hohman, Angela Jefferson, Lori L. Beason-Held, Susan M. Resnick, The Alzheimer’s Disease Neuroimaging Initiative (ADNI), The BIOCARD Study Team, Eleftherios Garyfallidis, Adam Anderson, Kurt G. Schilling, Bennett A. Landman, Daniel Moyer

**Affiliations:** a Department of Computer Science, Vanderbilt University, Nashville, TN, USA; b Department of Biomedical Engineering, Vanderbilt University, Nashville, TN, USA; c Vanderbilt Memory and Alzheimer’s Center, Vanderbilt University Medical Center, Nashville, TN, USA; d Department of Neurology, Vanderbilt University Medical Center, Nashville, TN, USA;; e Vanderbilt Genetics Institute, Vanderbilt University School of Medicine, Nashville, TN, USA; f Department of Medicine, Vanderbilt University Medical Center, Nashville, TN, USA; g Laboratory of Behavioral Neuroscience, National Institute on Aging, National Institutes of Health, Baltimore, MD, USA; h Intelligent Systems Engineering, Indiana University, Bloomington, IN, USA; i Department of Radiology and Radiological Services, Vanderbilt University Medical Center, Vanderbilt University Medical, Nashville, TN, USA; j Vanderbilt University Institute for Imaging Science, Vanderbilt University Medical Center, Nashville, TN, USA

**Keywords:** T1-weighted images, bias field correction, inhomogeneity, N4ITK, 3D U-Net

## Abstract

T1-weighted (T1w) MRI has low frequency intensity artifacts due to magnetic field inhomogeneities. Removal of these biases in T1w MRI images is a critical preprocessing step to ensure spatially consistent image interpretation. N4ITK bias field correction, the current state-of-the-art, is implemented in such a way that makes it difficult to port between different pipelines and workflows, thus making it hard to reimplement and reproduce results across local, cloud, and edge platforms. Moreover, N4ITK is opaque to optimization before and after its application, meaning that methodological development must work around the inhomogeneity correction step. Given the importance of bias fields correction in structural preprocessing and flexible implementation, we pursue a deep learning approximation / reinterpretation of the N4ITK bias fields correction to create a method which is portable, flexible, and fully differentiable. In this paper, we trained a deep learning network “DeepN4” on eight independent cohorts from 72 different scanners and age ranges with N4ITK-corrected T1w MRI and bias field for supervision in log space. We found that we can closely approximate N4ITK bias fields correction with naïve networks. We evaluate the peak signal to noise ratio (PSNR) in test dataset against the N4ITK corrected images. The median PSNR of corrected images between N4ITK and DeepN4 was 47.96 dB. In addition, we assess the DeepN4 model on eight additional external datasets and show the generalizability of the approach. This study establishes that incompatible N4ITK preprocessing steps can be closely approximated by naïve deep neural networks, facilitating more flexibility. All code and models are released at https://github.com/MASILab/DeepN4.

## 1. INTRODUCTION

Structural magnetic resonance imaging (MRI) highlights differences in tissue contrast based on the longitudinal relaxation time of hydrogen protons, making structural images suitable for delineating anatomical structures, abnormalities, and tissue types^1–3^. Clinically, structural images are frequently utilized as a reference to monitor the progression of disease and the efficacy of treatments for neurological disorders^4^. However, structural MRI suffers from intensity inhomogeneity artifacts appearing as a low frequency spatial intensity changes (“bias field”) that occur in part due to imperfections in the magnetic fields^5^. Correcting for these low frequency artifacts is a necessary preprocessing step in image processing. This helps avoid erroneous results in downstream analyses such as image segmentation, registration, texture analysis, and tissue classification^6,7^.

**Figure 1:**
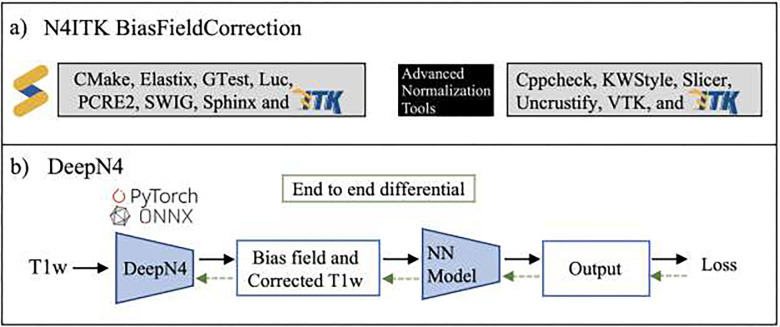
T1w MRI scans show spatial variations of image intensities, known as bias field effects, caused by the field inhomogeneity. a) The state-of-the-art framework that models the bias field has external dependencies that complicate integration into imaging pipelines. b) To address this, we propose DeepN4, a deep learning differentiable end-to end-model that utilizes the PyTorch python library; ONNX allows conversion across a deep learning framework. Our model allows loss function based on post-inhomogeneity correction. Our approach allows for the loss function based on post-inhomogeneity correction. Abbreviation: NN, Neural network.

There are several frameworks for eliminating the spatially varying bias fields^5,8^. In general, they follow two steps: (1) estimation of the bias field and (2) computing the corrected debiased image. Traditional correction methods can be classified as prospective^5,9–12^ or retrospective approaches^8^, with retrospective approaches gaining dominance due to their generalizability, efficiency, and fewer assumptions about the acquisition process^5^. Retrospective approaches use acquired images containing anatomical and intensity inhomogeneity information, along with prior knowledge of the imaging object. Retrospective approaches are further divided into filtering^13^, surface fitting, segmentation^14^, and histogram^15^ stages. Of particular significance to our work is the local histogram-based N3 method, which iteratively estimates the smooth multiplicative field by maximizing the high frequency component of the image intensity distribution^15^. Our method estimates this spatially varying multiplicative low frequency component with a deep learning network based on the principles of the N3 approach. A refinement of N3 is N4ITK which estimates the field at each iteration using the results of the previous iteration along with a *B*-spline approximation^16^, and is widely accepted due to the effectiveness and efficiency of the approach. N4ITK, provided by ITK, is integrated into various neuroimaging analysis tools, including SimpleITK^17^, ANTs^18^, FreeSurfer^19^, fmriPrep^20^, NiPype^21^, NeuroNorm^22^, and MRtrix^23^. Thus, in the past decade N4ITK has been recognized as the state-of-the-art (SOTA) approach and so we use N4ITK as a starting point for our own model design. Configuring ITK can be challenging, especially with the unfamiliarity with CMake, and requires careful consideration of compatibility across ITK, operating systems, compiler versions, and hardware platforms. While ITK is integrated into various neuroimaging analysis tools and is accessible to the public, the compilation process entails the installation of accompanying libraries and software packages. However, in cases where sole interest lies in N4 correction, these additional libraries become superfluous. For example, SimpleITK^17^ encompass Elastix, GTest, Luc, PCRE2, SWIG, and Sphinx, whereas ANTs^18^ extends to comprise Cppcheck, KWStyle, Slier, Uncrustify, and VTK, alongside ITK. This introduces complexities in terms of licensing and integration (Figure 1a).

The use of differentiable approaches for end-to-end learning pipelines is an actively evolving area of research^24,25^. Downstream tasks such as segmentation are often performed after inhomogeneity correction. However, optimization of parameters before inhomogeneity correction for outcomes measured afterwards is not easily done; N4ITK is opaque to gradient based optimization. Our paper addresses this problem by constructing an intermediate inhomogeneity correction step that is differentiable to optimize models before and after inhomogeneity correction. This enables the use of loss function based on the characteristics after inhomogeneity correction (Figure 1b).

Recently, deep learning models have achieved SOTA results in medical image processing tasks. Researchers have proposed deep learning-based methods for bias field correction of MR images^6,26–31^. Of these, there are two open source sharable models that fit our criteria, and are a feedforward CNN^6,26^ and an autoencoder^29^. Simkó et al. (2022) used implicit training on convolution neural network (CNNs) for bias field correction^26^. Sridhara et al. (2021) used an autoencoder based deep learning architecture to predict bias field that outperforms the conventional N4ITK approach^29^.

In the present work we provide a simple feedforward deep learning network for estimating the multiplicative field, trained with a direct (non-adversarial) loss term. We show parity or improvement on other open source models, and at a sufficiently high fidelity that further innovation and complexity seem unnecessary^6^. Thus, we propose a differentiable approach that estimates the smooth bias field while facilitating flexible and portable implementations of the SOTA N4ITK bias field correction from raw T1-weighted (T1w) MRI without complexity (Figure 1). The model is trained on a large repository of T1w images (Table 1) and then validated on eight external datasets (Table 2) to understand model performance on how the model estimated the low spatial frequency fields from high spatial frequency T1w MRI. Finally, we release and open source all model weights and inference scripts, allowing DeepN4 to be seamlessly integrated into other workflows.

## 2. METHODS AND MATERIALS

We model each bias field as a multiplicative field^16^. Rewriting Eqn. 1 from N3 paper^15^, we have

(1) a(r)=u(r)b(r)

where a is the acquired image, u is the corrected image, b is the bias field, and r is the voxel position of the images. We assume both u(r) and b(r) are greater than zero at all points r. Applying a logarithmic transformation and solving for corrected image, Eqn. 1 can be rewritten as log(u(r))=log(a(r))-log(b(r)). We aim to estimate log(b(r)) using a ral network. The following sections describe how our computing object was constructed, implemented, and trained. This is followed by an overview of the data used.

### 2.1. DeepN4 architecture

We parameterize the log-transformed bias field by a neural network, i.e.:

(3) $$logb^{\prime }(r)=f(a(r))$$

where function f is *DeepN4*, a generic 3D U-Net network and $b^{\prime }$ is the predicted bias field image. DeepN4 is a 3D U-Net framework based on the traditional architecture proposed by Ronneberger et al.^32^. The modification made in a well-validated three-dimensional image synthesis network^33^ was adapted in this network. It uses Leaky ReLU as activation function and instance batch normalization. The expanding path consists of corresponding transpose convolution layers to regain the spatial dimension of the input image. The convolution and devolution layers’ kernels are of size 3 × 3 × 3. The feature maps from the paths are concatenated via skip connections to retain both high-level and low-level features and enhance the accuracy of the model output.

**Figure 2:**
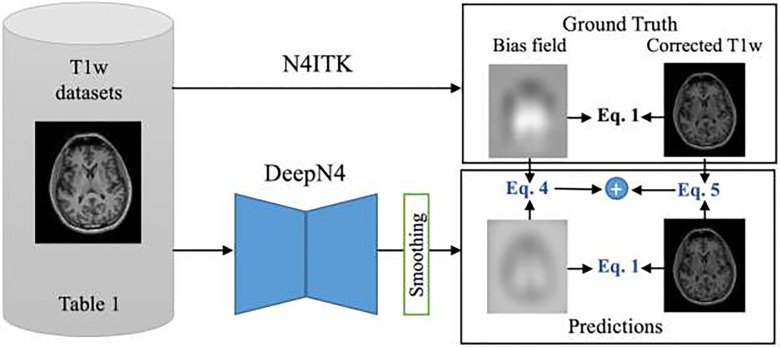
N4ITK was processed on the large-scale datasets in Table 1 to generate the ground truth bias field and corrected T1w images. All the T1w images in Table 1 were fed into the DeepN4 which outputs the log of predicted bias field. Smoothing is performed on predicted bias field from which the corrected image is obtained. The loss is minimized between the ground truth bias field and corrected T1w image with the predicted bias field and computed corrected T1w image using Eq. 4 and Eq. 5.

Upon obtaining the predicted bias field $e^{b^{\prime }(r)}$, we apply smoothing on the predicted bias field $e^{b^{\prime }(r)}$ (after the voxel-model) for further refinement and accuracy (Figure 2). There are two possible variations of smoothing. The first is a parameterized reconstruction employing *B*-splines to impose smoothness using *B-*spline functions. *B*-spline functions have local support and are numerically stable, making them a powerful tool for smoothing. The *B*-spline approximation from N4ITK is a uniform multivariate *B*-spline object of arbitrary order with resolution increasing at each successive level in the iteration process^16^. We perform slicewise smoothing using the B-spline model in ANTsPy^34^, which is a wrapper of the ITK B-spline approximation from ITKN4. B-spline was configured with a spline order of 3 and five fitting levels. Alternatively, a second option is approximating the smoothing with an isotropic. We choose a filter (kernel size 19 × 19 × 19 voxels) with standard deviation of 3 voxels which blur the bias field slightly^26^. Thus, the experiments can be summarized as DeepN4 NS, DeepN4 B, and DeepN4 G for no smoothing, *B*-spline, or Gaussian smoothing based on the smoothing approach after the U-Net architecture.

The loss function for DeepN4 is defined as $ℒ={ℒ}_{a}+{ℒ}_{b}$ where ${ℒ}_{a}$ is L2 loss function between the predicted and the ground truth bias fields, and ${ℒ}_{b}$ is L2 loss between the corrected image from the predicted bias field and the ground truth corrected image. That is:

(4) $${ℒ}_{a}=\frac{1}{N}\sum_{r=1}^{N}{\left(e^{\log b^{\prime }\left(r\right)}-b^{\prime }\left(r\right)\right)}^{2}$$

(5) $${ℒ}_{b}=\frac{1}{N}\sum_{r=1}^{N}{\left(e^{\text{log}\;a\left(r\right)-\text{log}\;b^{\prime }\left(r\right)}-u\left(r\right)\right)}^{2}$$

In Eqns. (4) and (5), N denotes the total number of masked voxels in image. The log predicted bias field b(r) is only computed within a brain mask to avoid background intensities of zero. The acquired image is divided by the smoothed bias field $e^{b^{\prime }(r)}$ to obtain the corrected image $u^{\prime }(r)$ (Figure 2).

Furthermore, we compared our proposed DeepN4 models to other open source deep learning based bias field correction methods. Specifically, we included the autoencoder model trained by Sridhara et al. using synthetic data from the HCP dataset, which is accessible at https://github.com/Shashank-95/Bias-Field-correction-in-3D-MRIs^29^ and the CNN model implicitly trained on images from BrainWeb by Simkó et al., which is available at https://github.com/attilasimko/bfc^26^.

### 2.2. Training protocol

For the training process, the network is optimized to minimize the loss function in section 2.2 using the Adam optimizer^35^ with a learning rate of 0.0001. We trained the model on a NVIDIA-Quadro RTX 5000 GPU with 16GB of memory. The model was trained on the training cohort with the ground truth consisting of the bias field and the corrected T1w image from N4ITK (Figure 2). The trained model that performed the best on the validation cohort was chosen to evaluate the test cohort.

### 2.3. Data overview

The objective of this study was to train a neural network model with diverse datasets obtained from different scanners with different resolutions, and different field strengths to create a robust and generalizable model approximating N4ITK, allowing the model to effectively handle variations in imaging protocols and produce accurate results. Consequently, we use de-identified data from eight distinct datasets as listed in Table 1 each with varying subjects, sessions, and scanner vendors. The ADNI cohort (https://adni.loni.usc.edu) began in 2003 as a public-private partnership, led by Principal Investigator, Michael W. Weiner, MD^36^. The NACC cohort began in 1999 and is comprised of dozens of Alzheimer’s Disease Research Centers that collect multimodal AD data^37^. The overall intention of the NACC cohort is to collate a large database of standardized clinical/neuropathological data^38–41^. There was a total of 10,424 T1w images that we randomly split into 90/5/5% as training, validation, and testing cohorts respectively. These were down-sampled to 2 × 2 × 2 mm and padded such that the image dimensions were 128 × 128 × 128 across all scans. The scans are normalized with min-max normalization, $\left(X-X_{min}\right)/\left(X_{max}-X_{min}\right)$ where $X_{max}$ is the 99^th^ intensity percentile of image X and $X_{min}$ is 0. The normalized image values are clipped between [0,1]. These down-sampled, padded, and normalized images are then used as input to the network discussed in this next section. For external validation, we used an additional set of eight external datasets (from sites distinct to those in Table 1), as outlined in Table 2. Seven of these eight datasets are publicly accessible. We evaluated the performance on DeepN4 NS, DeepN4 B, DeepN4 G, Sridhara et al.^29^, and Simkó et al.^26^ models on withheld and external test dataset in Table 1 and Table 2 respectively.

## 3. RESULTS

### 3.1 Quantitative performance

**Figure 3:**
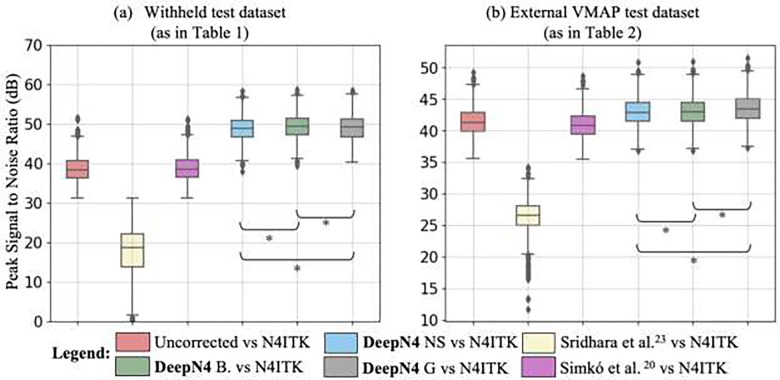
For both (a) and (b) DeepN4 models outperform existing models, and the reconstructed image is similar to state-of-the-art N4ITK. Higher PSNR indicates that reconstructed images from DeepN4 models are closer to N4ITK. The observed difference in DeepN4 B and DeepN4 G is effectively the negligible. DeepN4 NS = DeepN4 with no smoothing, DeepN4 G = DeepN4 with Gaussian smoothing, DeepN4 NS = DeepN4 with *B*-spline smoothing, and * *p* < 0.0001 (Wilcoxon sign rank test with Bonferroni correction).

To evaluate the performance of the proposed model, we computed the peak signal to noise ratio (PSNR) between N4ITK and DeepN4 corrected T1w images for (a) withheld test dataset of 1042 subjects and (b) external test dataset of 1074 imaging sessions (VMAP dataset), as shown in Figure 3. In the withheld test set as in Table 1, we find the median PSNR for images generated by the DeepN4 NS model was 48.96 dB, for DeepN4 B model was 49.38 dB, and for DeepN4 G was 49.23 (Figure 3a). For the subjects in VMAP (external) dataset as in Table 2, we find that the median PSNR for the DeepN4 NS model was 42.71 dB, for DeepN4 B was 42.87 dB, and DeepN4 G was 43.43 dB (Figure 3b). We observe that the DeepN4 models outperforms the existing Sridhara et al.^29^ and Simkó et al.^26^ with notable increase in the median PSNR of 23.21 and 3.71 dB respectively in withheld dataset. This indicates that DeepN4 model with access to a large and diverse dataset was able to generalize from the training set while the existing models with limited numbers of training data were not generalizable. Please note that the results from the models were mean shifted to uncorrected T1w image. This adjustment was made to compensate for the global intensity scaling in N4ITK, as the rescale option, which prevents intensity drift at each iteration, was not enabled on by default.

Additionally, we find that accuracy with Gaussian approximation (DeepN4 G) is 0.5 dB higher than *B-*spline regularization (DeepN4 B) on the external datasets and equivalent on the withheld data. This suggests the straightforward Gaussian approximation can serve as viable substitute for more resource-intensive *B*-spline regularization, which requires ANTsPy^34^ package. However, one potential factor contributing to this difference could be the higher number of randomly selected points used in Gaussian smoothing compared to B-spline.

The p-value between DeepN4 models was less than 0.0001 with a Bonferroni correction indicating that there was a statistically significant difference between the withheld dataset and the external dataset, reflecting the very large sample size and statistical power to detect small effects. We computed Cohens d, and the effect size was < 0.2 (considered small) between the models. This suggest DeepN4 models performed with similar effectiveness.

### 3.2 Qualitative performance

**Figure 4.**
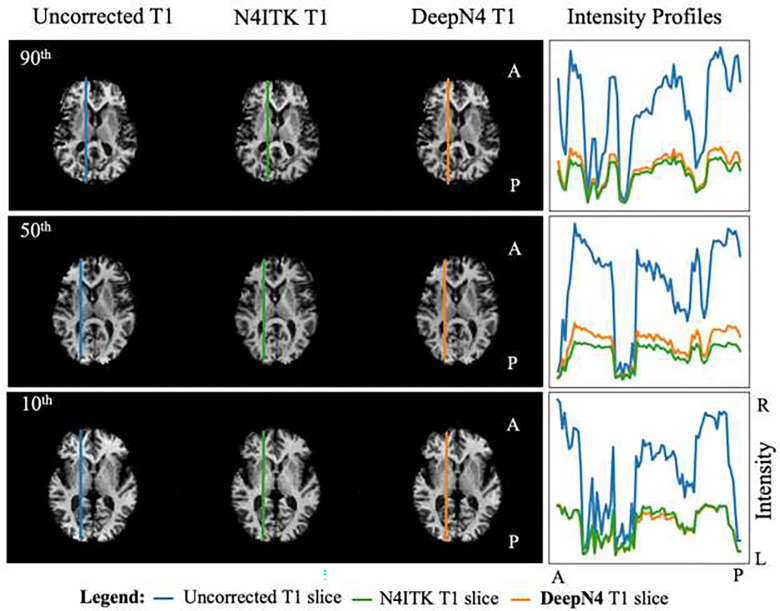
90^th^, 50^th^, and 10^th^ percentile sample are taken from DeepN4 G results in external VMAP dataset. Lower curvature between the intensity along a slice from uncorrected T1w (blue line), N4ITK corrected T1w (green line), and DeepN4 corrected T1w (orange line) denotes more uniformity. The intensity distribution along the slice in DeepN4 and N4ITK have no significant variation in performance across the 90^th^, 50^th^, and 10^th^ percentiles sample. A=Anterior, P=posterior.

Figure 4 shows the visualization of the results from 90^th^, 50^th^ and 10^th^ percentile images with respect to the DeepN4 G results in the external VMAP dataset (Figure 3 (b) gray) along with intensity profiles for a selected slice. We observe visually (1) noticeable inhomogeneity correction from T1w to N4ITK and (2) DeepN4 corrected T1w are similar to N4ITK T1w images. The intensity profiles of the slices highlighted in blue and orange from uncorrected T1w and DeepN4 corrected T1w respectively, indicate reduction in intensity non-uniformity. Our proposed method is effective at reducing the inhomogeneity.

**Figure 5:**
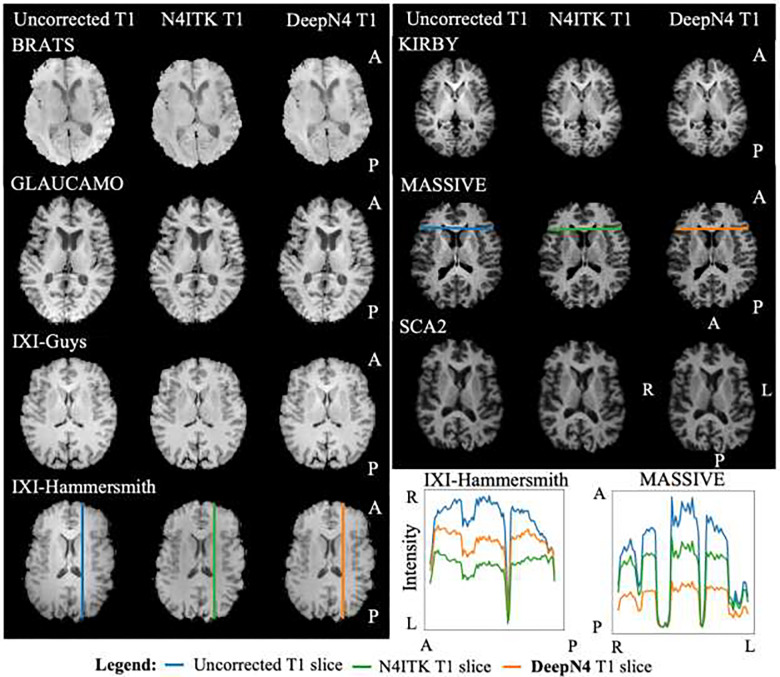
Here, we show DeepN4 results plotted against ITKN4 and the original T1w. DeepN4 results are similar to the ground truth N4ITK (SOTA, but neither easily accessible nor differentiable). Less curvature in the intensity of the selected slices in DeepN4 T1w (orange line) when compared to the uncorrected T1w slice (blue line) is more homogeneous. A=Anterior, P=posterior

To demonstrate generalizability, we apply our model across external independent datasets in Table 2. Here, we show the resulting images from the sample which had median PSNR value between DeepN4 G and N4ITK in each of the external dataset (Figure 5). In all cases, we find the DeepN4 corrected images are similar to the SOTA N4ITK. This suggests the model is well generalizable to images from different cohorts with different characteristics. Note that for Figure 4 and 5 the DeepN4 results are shown with Gaussian smoothing since the results in Figure 3 show that performance with Gaussian smoothing and B-spline smoothing are essentially identical.

## 4. CONCLUSION

Adapting the state-of-the-art N4ITK bias field correction in model pipelines is challenging due to its intricate dependency stack. It is not possible to have end-to-end differentiable segmentation models using ITKN4. In this work, we address these concerns by training DeepN4, a generic 3D U-Net with loss functions based on the principals of N4ITK. Thus, we make inhomogeneity correction transparent and amenable to optimization.

Although researchers have proposed various novel deep learning frameworks for bias field correction of MR images, such as BiasNet^6^, implicit training on CNNs^26^, reconstruction algorithms^31^, and deep learning networks based on generative adversarial net^30^, these approaches have lacked flexibility and generalizability. The proposed simple technique has shown the feasibility of estimating and eliminating low frequency inhomogeneities on to rigid high frequency anatomical structures with naïve 3D networks. Our experiments show (1) similar performance to N4ITK both qualitatively and quantitatively and (2) consistent performance on multiple, independent, unrelated external datasets, indicating the generalizability of our model. Thus, our model is easily understandable, efficient in performance, and readily available to incorporate into any existing pipeline with the inference function.

Deep learning models have employed *B*-spline for data augmentation^54^. A potential direction for future research is to explore the integration of a *B*-spline layer within the neural network and having an iterative network approach.

The pretrained pipeline is available in the form of a container along with source code and can be accessed by following the instructions at https://github.com/MASILab/DeepN4.

## Figures and Tables

**Table 1.** Datasets used for training, validation, and testing of DeepN4.

Dataset	Subjects	Sessions	T1w Images	Vendor (Scanner)	Field Strength	Resolution (mm)
ADNI36	799	1–5	1905	Siemens, GE, and Philips (61)	3T	1 iso 1.2x1x1 1.2x1.054x1.054 2x1x1
BLSA^42^	1151	1–8	2869	Philips (3)	3T	1.2x1x1
OASIS-3^43^	992	1–6	2452	Siemens (2)	1.5T,3T	1x1x1 1.2x1x1
NACC	273	1–2	288	Philips (1)	3T	1.2x1x1
BIOCARD^44^	212	1–4	508	Philips (1)	3T	1.2x1x1
HCP YA^45^	1112	1	1112	Siemens (1)	3T	0.7 iso
HCP Aging^46^	664	1	664	Siemens (1)	3T	0.8 iso
HCP Dev^46^	626	1	626	Siemens (1)	3T	0.8 iso
Learning (Training + Validation )	-	-	9382 (8340+ 1042)	-	-	2 iso
Testing (Withheld)	-	-	1042	-	-	2 iso

iso = isotropic, - = not applicable

**Table 2.** Datasets used for external validation of DeepN4.

Dataset	Subjects	Sessions	T1w Images	Vendor (Scanner)	Field Strength	Resolution (mm)
VMAP^47^	327	1–4	1074	Philips (1)	3T	1 iso
KIRBY^48^	5	5	5	Philips (1)	3T	1.2x1x1
SCA2^49^ (ds001378)	5	5	5	Philips (1)	1.5T	1 iso
IXI Hammersmith^50^	5	5	5	Philips (1)	3T	1.2x1x1
IXI Guys^50^	5	5	5	Philips (1)	1.5T	1.2x1x1
MASSIVE^51^	1	5	5	Philips (1)	3T	1 iso
BRATS^52^	5	5	5	Siemens, GE, and Philips (5)	1.5T,3T	1 iso
GLAUCAMO^53^ (ds001743)	5	5	5	GE (1)	3T	1 iso

iso = isotropic

## Data Availability

The data used for the analysis of this article are confidential due to privacy or other restrictions.
